# Rathke's Cleft Cyst as Origin of a Pediatric Papillary Craniopharyngioma

**DOI:** 10.3389/fgene.2018.00049

**Published:** 2018-02-22

**Authors:** Sven-Martin Schlaffer, Michael Buchfelder, Robert Stoehr, Rolf Buslei, Annett Hölsken

**Affiliations:** ^1^Department of Neurosurgery, University Hospital Erlangen, Friedrich-Alexander University Erlangen-Nürnberg, Erlangen, Germany; ^2^Institute of Pathology, University Hospital Erlangen, Friedrich-Alexander University Erlangen-Nürnberg, Erlangen, Germany; ^3^Institute of Neuropathology, University Hospital Erlangen Friedrich-Alexander University Erlangen-Nürnberg, Erlangen, Germany; ^4^Institute of Pathology, Sozialstiftung Bamberg, Bamberg, Germany

**Keywords:** Rathke's cleft cyst, *BRAF* mutation, metaplasia, papillary craniopharyngioma, childhood

## Abstract

A 6-year old patient presented with an intra and suprasellar cystic lesion accompanied with impairment of the hypothalamic-pituitary axis and partial hypopituitarism. The most likely cause of sellar lesions in this age group are adamantinomatous craniopharyngioma (adaCP) or Rathke´s cleft cysts (RCCs). AdaCP are characterized by *CTNNB1* mutations accompanied with aberrant nuclear beta-catenin expression. RCC show neither nuclear beta-catenin expression nor *BRAF* mutation. The latter is a hallmark of papillary craniopharyngiomas (papCP) that exhibit remarkable histological similarity with metaplasia of RCC. Diagnosis of the patient was elucidated by *CTNNB1* and *BRAF* mutation screening, utilizing different approaches, as well as histological examination of markers, e.g., beta-catenin, claudin-1, EpCAM and the mutated BRAFV600E protein, which are known to be differentially expressed in sellar lesions. The case presented reveals extraordinary aspects for two reasons. Firstly, the lesion appeared clinically, on MRI, intraoperatively and histologically as RCC with prominent squamous metaplasia, but showing an expression pattern of markers also found in papCP, whilst exhibiting a hitherto undescribed *BRAF*^*V*600*E*^ mutation. This important result documents a supposable transition of RCC metaplasia into a papillary craniopharyngioma (papCP). Secondly, this intriguing case shows unexpectedly that although papCP usually occurs almost exclusively in adults, it can also arise in childhood.

## Introduction

**Figure 1:**
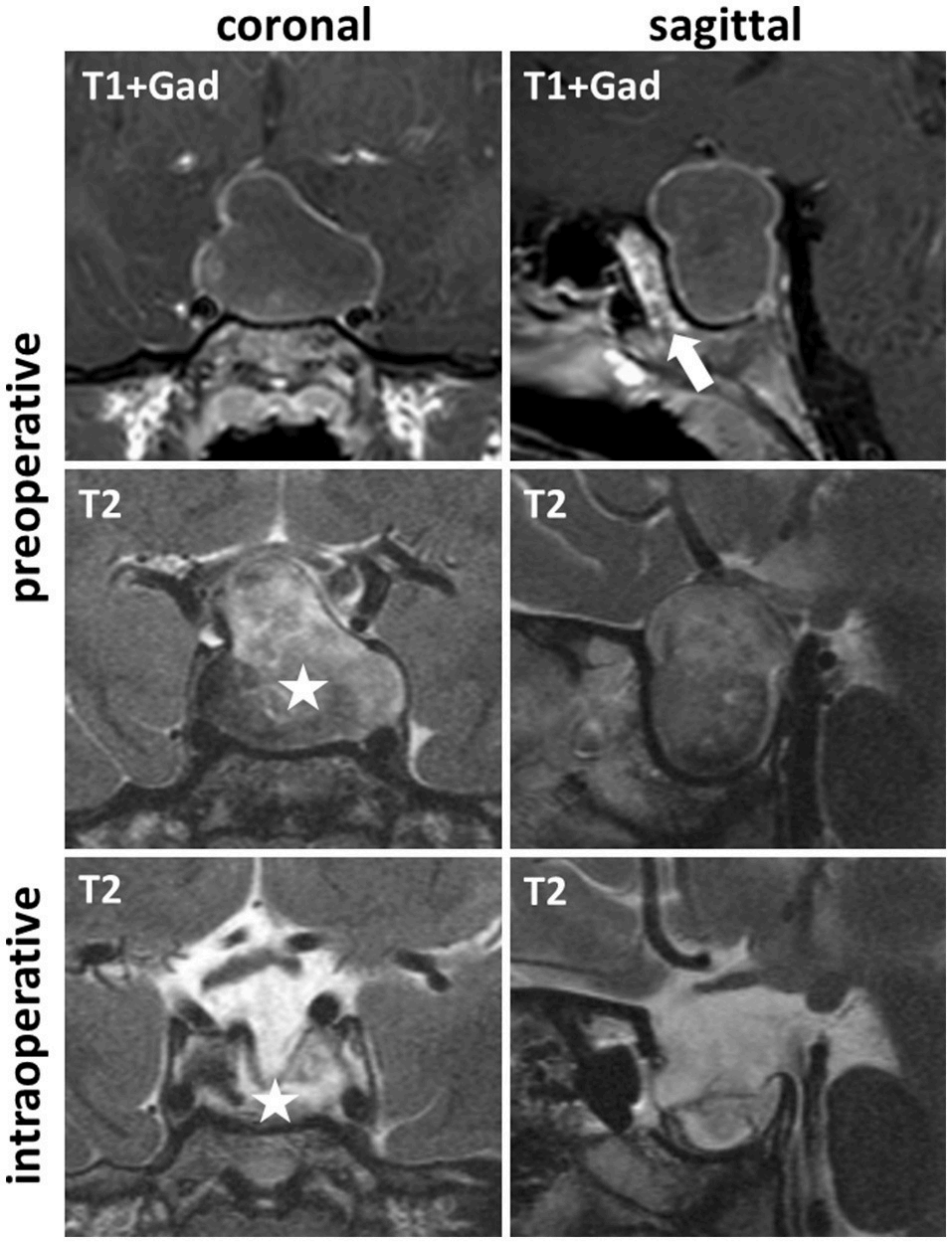
Pre- and intraoperative MRI; T1-weighted MR images after gadolinium contrast medium application (T1+Gad) and T2-weighted MRI showed in coronal and sagittal orientation a preoperative intra and suprasellar cystic tumor, lacking sphenoid sinus pneumatization (T1+Gad, sagittal, arrow). Intraoperative imaging confirmed complete drainage of the cyst (T2 coronal, asterisk).

A 6 year old child progressively developed polydipsia and polyuria with a fluid intake of more than 4 l per day since April 2016. Interestingly, the child was hospitalized for several days only 1 month prior in another hospital with abacterial meningitis without any laboratory abnormalities. Laboratory tests revealed a central diabetes insipidus and the MRI displayed an intra and suprasellar, cystic, slightly contrast enhancing mass (Figure 1). Further endocrinological evaluation indicated a slightly elevated prolactin level and a thyroid and somatotrophic insufficiency resulting in short stature (106 cm, 16 kg; both below 3rd percentile). In addition to the MRI, a CT-scan showed no calcifications of the 26 × 18 × 26 mm cystic tumor with light compression of the optic chiasm.

## Background

The most common cystic intrasellar lesions with suprasellar extensions in the pediatric population are adamantionomatous craniopharyngiomas (adaCP), accounting for nearly 50% of sellar lesions in childhood, and Rathke´s cleft cysts (RCC) (Schroeder and Vezina, 2011). Cysts of adaCP are filled with a viscous machine oil-like fluid containing cholesterol crystals and cell debris. However, solid components of the lesion are typically comprised of calcifications and wet keratin interspersed between epithelial components (Louis et al., 2016). The latter are characterized by activating mutations in the *CTNNB1* gene encoding beta-catenin (Sekine et al., 2002; Buslei et al., 2005). The immunohistochemically evident cytoplasmic and nuclear accumulation of beta-catenin in distinct cells represents a diagnostic hallmark of adaCP, setting them apart from other sellar lesions, e.g., RCC or papillary craniopharyngiomas (papCP) (Buslei et al., 2005).

RCC filled with a protein containing fluid arise from the pars intermedia between the anterior and posterior lobe of the pituitary gland. Although RCC are diagnosed in pediatric patients, this is a generally rare event (Fan et al., 2012; Trifanescu et al., 2012). Up to 87% of the cysts show suprasellar enlargement (Schroeder and Vezina, 2011). The cyst wall is composed of ciliated columnar or cuboidal epithelium containing goblet cells (Schweizer et al., 2015). It is worth noting that in 5–17% of RCC cases, squamous metaplasia is observed (Potts et al., 2011), which exhibits striking histological similarities with the well differentiated non-keratinizing squamous epithelium of papCP (Louis et al., 2016), thereby often rendering histological classification challenging. This second, also benign craniopharyngioma subtype, occurs less frequently than adaCP and is characterized by a *BRAF* mutation (Brastianos et al., 2014) resulting in an up to 500-fold increased activation of v-raf murine sarcoma viral oncogene homolog B1 (BRAF) (Cantwell-Dorris et al., 2011). BRAF regulates the conserved RAS-RAF-MEK-ERK (MAPK) pathway as an intermediate, controlling important cellular processes, e.g., proliferation, differentiation and survival. Interrupting excessive signaling in recurrent papCP provides a promising therapeutic option (Aylwin et al., 2015; Brastianos and Santagata, 2016; Brastianos et al., 2016). Based on the histological similarity of both entities, studies have been initiated to determine if papCP arise from squamous metaplasia of RCC. However, *BRAF* mutational analysis was identified as a reliable tool for distinguishing both lesions in recent studies, as RCC with squamous metaplasia show no *BRAF* mutation (Kim et al., 2015; Marucci et al., 2015; Schweizer et al., 2015). It is worth noting that four cases of previously diagnosed RCC were re-classified as papCP because they were *BRAF*^*V*600*E*^ positive (Marucci et al., 2015; Schweizer et al., 2015). This illustrates the level of difficulty involved in histological evaluation and implies a transformation of RCC into papCP, which emphasizes the importance of mutational analysis for correct grading. In contrast to adaCP which are particularly prevalent (Louis et al., 2016) in children but also in adulthood, “papCP occur almost exclusively in adults …” as stated in the new WHO Classification of Tumors of the Central Nervous System (Louis et al., 2016).

## Discussion

### Surgical treatment and path to diagnosis

Since the sphenoid sinus of the 6-year-old child was not pneumatized, a surgical approach and resection was performed with neuronavigation guidance and intraoperative MRI for resection control.

After a microsurgical, sublabial, paraseptal approach, the sphenoid bone was drilled until the basal sellar dura was widely exposed. After incision, a yellowish fluid was drained and the cyst completely collapsed. A thin membrane was identified and subtotally resected, leaving some remnants on the diaphragm sellae. Intraoperative MRI revealed a complete evacuation of the cystic component (Figure 1, asterisk). At the end of the procedure, minor CSF-leakage was detected and sealed with fibrin-glue and some facia lata of the right thigh.

The post-operative course was uneventful and no further endocrinological deficiency was detected. The patient was discharged 9 days post-surgery.

The intraoperative appearance of the cyst content with its viscous consistency, yellowish color and lack of the typical hallmarks of a craniopharyngioma-like cholesterol crystals and a thick, microcalcified membrane, raised the clinical diagnosis of an RCC rather than a craniopharyngioma.

**Figure 2:**
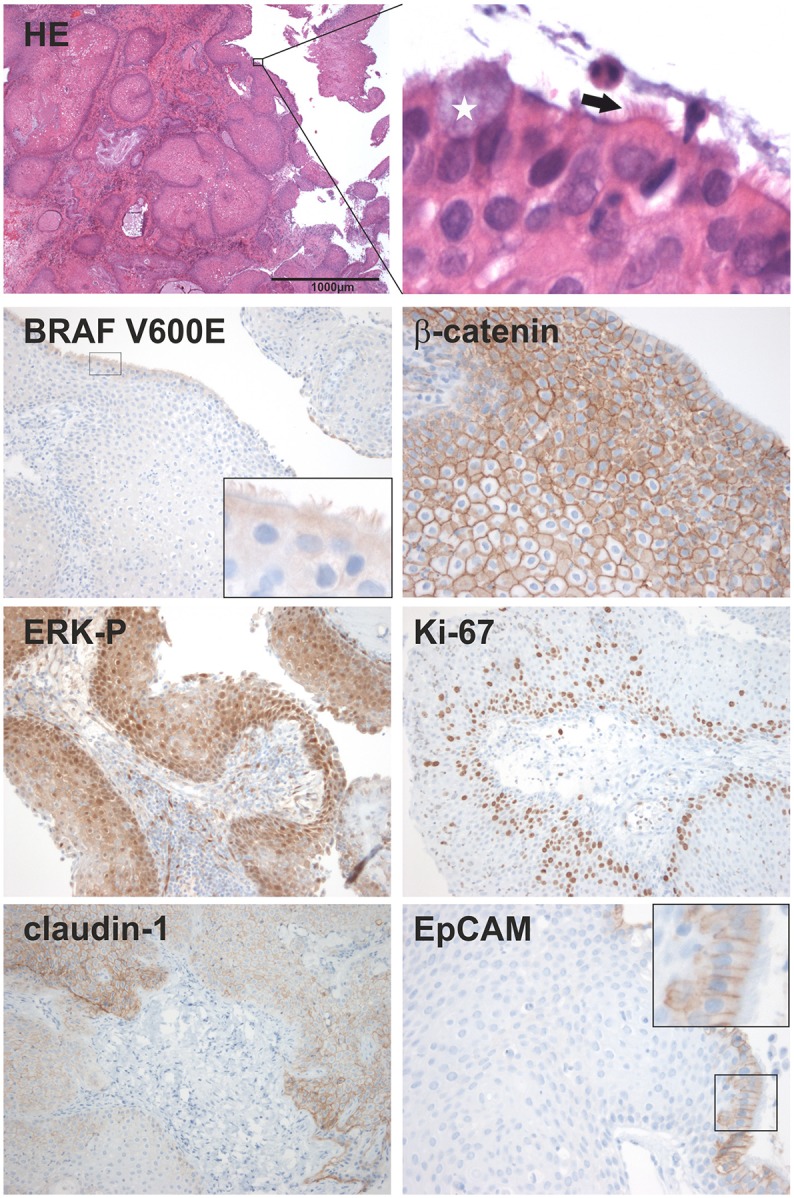
Histological characterization; HE staining shows a lesion of squamous couple stone differentiated epithelium. Magnification revealed a superficial cell layer harboring kinocilia (arrow) and goblet cells (white star). The following immunohistochemical stainings show the evidence and expression pattern of the mutated BRAF V600E protein, beta-catenin; the phosphorylated ERK (ERK-P) and the proliferation marker Ki-67; as well as the distribution of the cell-cell contact proteins, claudin-1 and EpCAM. In a higher resolution, EpCAM is only detectable in ciliated cells of the superficial cell layer.

Histologically, the resected lesion unites criteria of RCC with extensive squamous metaplasia possessing apical ciliated columnar epithelium and interspersed goblet cells (Figure 2, HE magnification) with those of a papCP, consisting of well differentiated squamous epithelium. The proliferation index reached focally up to 15% within the basal squamous epithelial cell layers (Ki-67, Figure 2). Further cell adhesion markers described as differentially expressed in RCC and CP subtypes were evaluated. The membranous expression of claudin-1 revealed a typical homogeneous staining pattern as described in papCP (Stache et al., 2014). The negative expression of EpCAM (epithelial cell adhesion molecule) in metaplastic squamous epithelium is congruent with observations in papCP, whereas ciliated epithelial cells are positive (Figure 2, magnification) as identified in RCC (Thimsen et al., 2016).

### *BRAF* mutation analysis reveals diagnosis of a papCP

**Figure 3:**
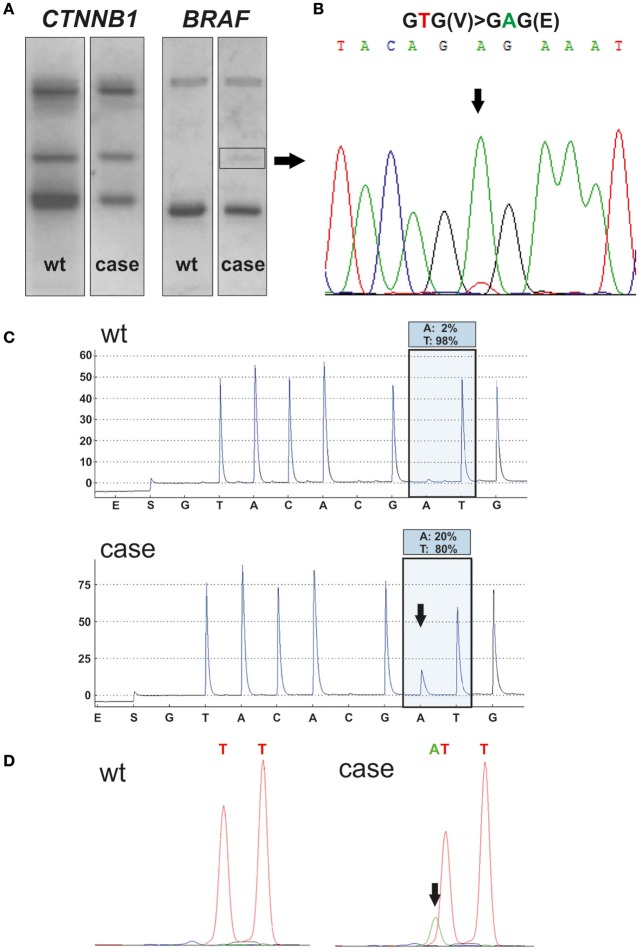
Mutational analysis; SSCP analysis of the hot spot region of *CTNNB1* (exon 3) and *BRAF* (exon 15) was performed using DNA extracted from the formalin fixed, paraffin embedded resected tissue of the child (case line) **(A)**. Wildtype DNA (wt line) was used as a control and to compare mutation induced aberrant bands. Only *BRAF* showed a shifted band (square), revealing in Sanger sequencing a *BRAF*^*V*600*E*^ mutation with a thymine (T) to adenine (A) transversion **(B)**. Pyrosequencing **(C)** confirmed *BRAF*^*V*600*E*^ mutation (allele frequency of A: 20%), compared to the wt control (allele frequency of A: 3%). Pyrosequencing was performed twice in the department of neuropathology and department of pathology with comparable results. SNapShot analysis **(D)** confirms thymine to adenine transversion (arrow) in relation to wt control, which leads to an amino acid exchange of valine to glutamic acid at codon 600 within the *BRAF* gene.

Immunohistochemical detection of the BRAFV600E mutant protein in the squamous epithelium using a mutation specific antibody (clone VE1) was inconclusive, although the labeling of ciliated cells must be interpreted as a false positive staining (Jones et al., 2015) (Figure 2). However, *BRAF*^*V*600*E*^ mutation (Figure 3) suggested a papCP diagnosis, which was identified and confirmed by mutation analyses carried out independently in two departments using various methods, e.g., single stranded conformation polymorphism (SSCP) with subsequent sanger sequencing (Figure 3B) of the shifted band (square, Figure 3A) (Holsken et al., 2016), Pyrosequencing (Holsken et al., 2016) and SNapShot analysis (Lurkin et al., 2010) (Figures 3C,D). Furthermore, the mutation derived BRAF activation of the MAPK cascade leads downstream to phosphorylation of ERK (extracellular signal-regulated kinase) functioning within the nucleus as an activator of transcription factors (Cantwell-Dorris et al., 2011), which was verified within the squamous epithelium (Figure 2).

For the sake of completeness, adaCP diagnosis, which is common in the pediatric age group, was excluded by the histomorphological appearance of the lesion and by beta-catenin analysis. Neither an immunohistochemically nuclear accumulation of beta-catenin (Figure 2; Buslei et al., 2005) nor mutations within exon 3 of the *CTNNB1*gene using SSCP (Figure 3A) and Sanger sequencing (data not shown) were detectable.

Furthermore, there was no histological evidence for Langerhans cell histiocytosis. This histiocytic tumor was excluded because it occurs mostly in childhood, could also be seen in the hypothalymic-pituitary region, and carries a *BRAF*^*V*600*E*^ mutation in 38–58% of cases (Louis et al., 2016).

## Concluding remarks

This is the first report describing a pediatric patient with a sellar mass lesion that appears clinically and histologically as an RCC with pronounced metaplastic squamous epithelium but harbors a *BRAF*^*V*600*E*^ mutation representing a hallmark of papCP. Therefore, this supports the assumption that papCP could originate from RCC. Additionally, this intriguing case unexpectedly shows that papCP can occur in childhood.

## Informed consent and ethical approval

The patients' parents/legal guardians provided written informed consent for research participation as well as for the publication of the child's indirectly identifiable information. All methods used were carried out in accordance with the approved guidelines of the Ethical Committee of the University of Erlangen-Nürnberg and in accordance with the Declaration of Helsinki.

## Author contributions

Conception and design: S-MS and AH. Providing clinical data: S-MS and MB. Histological and genetic analyses: AH, RS, and RB. Drafting the article: AH and S-MS. Critically revising the article: RB, MB, RS, and RB.

### Conflict of interest statement

The authors declare that the research was conducted in the absence of any commercial or financial relationships that could be construed as a potential conflict of interest.

## Abbreviations

RCC: Rathke´s cleft cysts
papCP: papillary craniopharyngioma
adaCP: adamantinomatous craniopharyngioma
BRAF: v-raf murine sarcoma viral oncogene homolog B1
EpCAM: epithelial cell adhesion molecule
ERK: extracellular signal-regulated kinase
SSCP: single stranded conformation polymorphism
CTNNB1: Catenin Beta 1.

## References

[B1] Aylwin S. J. Bodi I. Beaney R. (2015). Pronounced response of papillary craniopharyngioma to treatment with vemurafenib, a BRAF inhibitor. Pituitary 19, 544–546. 10.1007/s11102-015-0663-4 26115708 PMC4996872

[B2] Brastianos P. K. Santagata S. (2016). ENDOCRINE TUMORS: BRAF V600E mutations in papillary craniopharyngioma. Eur. J. Endocrinol. 174, R139–R144. 10.1530/EJE-15-0957 26563980 PMC4876601

[B3] Brastianos P. K. Shankar G. M. Gill C. M. Taylor-Weiner A. Nayyar N. Panka D. J.. (2016). Dramatic response of BRAF V600E mutant papillary craniopharyngioma to targeted therapy. J. Natl. Cancer Inst. 108:djv310. 10.1093/jnci/djv310 26498373 PMC4862417

[B4] Brastianos P. K. Taylor-Weiner A. Manley P. E. Jones R. T. Dias-Santagata D. Thorner A. R.. (2014). Exome sequencing identifies BRAF mutations in papillary craniopharyngiomas. Nat. Genet. 46, 161–165. 10.1038/ng.2868 24413733 PMC3982316

[B5] Buslei R. Nolde M. Hofmann B. Meissner S. Eyupoglu I. Y. Siebzehnrubl F. (2005). Common mutations of beta-catenin in adamantinomatous craniopharyngiomas but not in other tumours originating from the sellar region. Acta Neuropathol. 109, 589–597. 10.1007/s00401-005-1004-x 15891929

[B6] Cantwell-Dorris E. R. O'Leary J. J. Sheils O. M. (2011). BRAFV600E: implications for carcinogenesis and molecular therapy. Mol. Cancer Ther. 10, 385–394. 10.1158/1535-7163.MCT-10-0799 21388974

[B7] Fan M. C. Wang Q. L. Wang J. F. Deng W. S. Li L. D. Wang Z. H. (2012). Surgical treatment of symptomatic Rathke´s cleft cysts: clinical features, therapy considerations and outcomes. Chin. Med. J. 125, 2919–2924. 22932091

[B8] Hölsken A. Sill M. Merkle J. Schweizer L. Buchfelder M. Flitsch J.. (2016). Adamantinomatous and papillary craniopharyngiomas are characterized by distinct epigenomic as well as mutational and transcriptomic profiles. Acta Neuropathol. Commun. 4:20. 10.1186/s40478-016-0287-6 26927026 PMC4770705

[B9] Jones R. T. Abedalthagafi M. S. Brahmandam M. Greenfield E. A. Hoang M. P. Louis D. N.. (2015). Cross-reactivity of the BRAF VE1 antibody with epitopes in axonemal dyneins leads to staining of cilia. Modern Pathol. 28, 596–606. 10.1038/modpathol.2014.150 25412847

[B10] Kim J. H. Paulus W. Heim S. (2015). BRAF V600E mutation is a useful marker for differentiating Rathke´s cleft cyst with squamous metaplasia from papillary craniopharyngioma. J. Neurooncol. 123, 189–191. 10.1007/s11060-015-1757-6 25820214

[B11] Louis D. Ohgaki H. Wiestler O. Cavenee W. Ellison D. Figarella-Branger D. (2016). WHO Classification of Tumours of the Central Nervous System, Revised, 4th Edn. Lyon: IARC. 10.1007/s00401-016-1545-1 27157931

[B12] Lurkin I. Stoehr R. Hurst C. D. van Tilborg A. A. Knowles M. A. Hartmann A.. (2010). Two multiplex assays that simultaneously identify 22 possible mutation sites in the KRAS, BRAF, NRAS and PIK3CA genes. PLoS ONE 5:e8802. 10.1371/journal.pone.0008802 20098682 PMC2809099

[B13] Marucci G. de Biase D. Zoli M. Faustini-Fustini M. Bacci A. Pasquini E.. (2015). Targeted BRAF and CTNNB1 next-generation sequencing allows proper classification of nonadenomatous lesions of the sellar region in samples with limiting amounts of lesional cells. Pituitary 18, 905–911. 10.1007/s11102-015-0669-y 26156055

[B14] Potts M. B. Jahangiri A. Lamborn K. R. Blevins L. S. Kunwar S. Aghi M. K. (2011). Suprasellar Rathke cleft cysts: clinical presentation and treatment outcomes. Neurosurgery 69, 1058–1068. discussion: 1068–1067. 10.1227/NEU.0b013e318228bcea 21673610

[B15] Schroeder J. W. Vezina L. G. (2011). Pediatric sellar and suprasellar lesions. Pediatr. Radiol. 41, 287–298; quiz 404–405. 10.1007/s00247-010-1968-0 21267556

[B16] Schweizer L. Capper D. Hölsken A. Fahlbusch R. Flitsch J. Buchfelder M. (2015). BRAF V600E analysis for the differentiation of papillary craniopharyngiomas and Rathke´s cleft cysts. Neuropathol. Appl. Neurobiol. 41, 733–742. 10.1111/nan.12201 25442675

[B17] Sekine S. Shibata T. Kokubu A. Morishita Y. Noguchi M. Nakanishi Y.. (2002). Craniopharyngiomas of adamantinomatous type harbor beta-catenin gene mutations. Am. J. Pathol. 161, 1997–2001. 10.1016/S0002-9440(10)64477-X 12466115 PMC1850925

[B18] Stache C. Hölsken A. Fahlbusch R. Flitsch J. Schlaffer S. M. Buchfelder M.. (2014). Tight junction protein claudin-1 is differentially expressed in craniopharyngioma subtypes and indicates invasive tumor growth. Neuro. Oncol. 16, 256–264. 10.1093/neuonc/not195 24305709 PMC3895384

[B19] Thimsen V. Hölsken A. Buchfelder M. Flitsch J. Fahlbusch R. Stefanits H. (2016). EpCAM (CD326) is differentially expressed in craniopharyngioma subtypes and Rathke´s cleft cysts. Sci. Rep. 6:29731 10.1038/srep29731 27431859 PMC4949472

[B20] Trifanescu R. Ansorge O. Wass J. A. Grossman A. B. Karavitaki N. (2012). Rathke´s cleft cysts. Clin. Endocrinol. 76, 151–160. 10.1111/j.1365-2265.2011.04235.x 21951110

